# Potential Importance of Molybdenum Priming to Metabolism and Nutritive Value of *Canavalia* spp. Sprouts

**DOI:** 10.3390/plants10112387

**Published:** 2021-11-05

**Authors:** Mohammad K. Okla, Nosheen Akhtar, Saud A. Alamri, Salem Mesfir Al-Qahtani, Ahmed Ismail, Zahid Khurshid Abbas, Abdullah A. AL-Ghamdi, Ahmad A. Qahtan, Walid H. Soufan, Ibrahim A. Alaraidh, Samy Selim, Hamada AbdElgawad

**Affiliations:** 1 Botany and Microbiology Department, College of Science, King Saud University, Riyadh 11451, Saudi Arabia; saualamri@ksu.edu.sa (S.A.A.); abdaalghamdi@ksu.edu.sa (A.A.A.-G.); aqahtan@ksu.edu.sa (A.A.Q.); ialaraidh@ksu.edu.sa (I.A.A.); 2 Department of Biological Sciences, National University of Medical Sciences, Rawalpindi 46000, Pakistan; 3 Biology Department, University College of Taymma, Tabuk University, Tabuk 71411, Saudi Arabia; salghtani@ut.edu.sa; 4 Pharmacognosy Department, Faculty of Pharmacy, Fayoum University, Fayoum 63514, Egypt; Ais03@fayoum.edu.eg; 5 Department of Biology, Faculty of Science, University of Tabuk, Tabuk 71491, Saudi Arabia; Znourabbas@ut.edu.sa; 6 Department of Plant Production, Faculty of Food and Agriculture Sciences, King Saud University, Riyadh 11451, Saudi Arabia; waoufan@ksu.edu.sa; 7 Department of Clinical Laboratory Sciences, College of Applied Medical Sciences, Jouf University, Sakaka 72388, Saudi Arabia; sabdulsalam@ju.edu.sa; 8 Integrated Molecular Plant Physiology Research, Department of Biology, University of Antwerp, 2020 Antwerpen, Belgium; hamada.abdelgawad@uantwerpen.be; 9 Botany and Microbiology Department, Faculty of Science, Beni-Suef University, Beni-Suef 62511, Egypt

**Keywords:** *Canavalia*, molybdenum, seed priming, nitrogen assimilation

## Abstract

Molybdenum ions (Mo) can improve plants’ nutritional value primarily by enhancing nitrogenous metabolism. In this study, the comparative effects of seed priming using Mo were evaluated among sproutings of *Canavalia* species/cultivars, including *Canavalia ensiformis var. gladiata* (CA1), *Canavalia ensiformis var. truncata Ricker* (CA2), and *Canavalia gladiata var. alba Hisauc* (CA3). Mo impacts on growth, metabolism (e.g., nitrogen and phenolic metabolism, pigment and total nutrient profiles), and biological activities were assayed. Principal component analysis (PCA) was used to correlate Mo-mediated impacts. The results showed that Mo induced photosynthetic pigments that resulted in an improvement in growth and increased biomass. The N content was increased 0.3-fold in CA3 and 0.2-fold in CA1 and CA2. Enhanced nitrogen metabolism by Mo provided the precursors for amino acids, protein, and lipid biosynthesis. At the secondary metabolic level, phenolic metabolism-related precursors and enzyme activities were also differentially increased in *Canavalia* species/cultivars. The observed increase in metabolism resulted in the enhancement of the antioxidant (2,2-diphenyl-1-picryl-hydrazyl-hydrate (DPPH) free radical scavenging, 2,2-azino-bis(3-ethylbenzothiazoline-6-sulfonic acid) (ABTS), ferric reducing antioxidant power (FRAP)) and antidiabetic potential (Glycemic index (GI) and inhibition activity of α-amylase, and α-glucosidase) of species. The antioxidant activity increased 20% in CA3, 14% in CA1, and 8% in CA2. Furthermore, PCA showed significant variations not only between Mo-treated and untreated samples but also among *Canavalia* species. Overall, this study indicated that the sprouts of *Canavalia* species have tremendous potential for commercial usage due to their high nutritive value, which can be enhanced further with Mo treatment to accomplish the demand for nutritious feed.

## 1. Introduction

Developing countries are facing an increasing demand for protein and other nutrient-rich foods. In this context, legumes can contribute as the most valuable source of nutrients and provide high-quality dietary proteins [1]. Legume plants have desirable characteristics such as an abundance of carbohydrates, the ability to lower serum cholesterol, high fiber, a high concentration of polyunsaturated fatty acids, and long shelf life. In addition to B complex vitamins such as folate, thiamin, and riboflavin, minerals, and fiber, legumes are also major sources of proteins and calories [2]. Furthermore, it is evidenced that sprouts are the richest source of proteins and other compounds of nutritional value compared to un-sprouted plants [3]. Moreover, sprouts have also been associated with a variety of biologically active constituents with potential health benefits. During germination, metabolic enzymes are activated, which can lead to the release of some amino acids and peptides, and the synthesis or use of them can form new proteins. As a consequence, the nutritional and medicinal value might be enhanced by sprouting in legumes. Research has to be geared to exploit the sprouting of legumes and enhance their nutrition values to meet the nutritional requirements of the increasing population. 

The genus of *Canavalia* is considered the third largest family among flowering plants [1]. It comprises approximately 50 species of tropical vines widely distributed in tropical and subtropical regions all over the world [4]. This genus was used traditionally as a food due to its nutritional significance. Sridhar and Seena envisaged a comparative account of nutritional and functional properties of *Canavalia* species [5]. *Canavalia gladiata* and *Canavalia ensiformis* are the common legume species having the potential to be a rich protein source, like edible legumes [5]. Pharmacological effects of *Canavalia gladiata* are reported for cancer [6], allergies [7], antioxidants [8], and inflammation [9]. *Canavalia gladiata* in complex with *Arctium lappa* extract is proposed to develop as a functional food for stimulating immunity [10]. Similarly, the seeds of *Canavalia ensiformis* are a source of proteins with biotechnological importance including ureases [11] and proteases [12]. Processed seeds of *Canavalia ensiformis* are reported for enhanced antioxidant activity [13]. Hence, *Canavalia* species are of high medical importance, and with proper seed priming with micronutrients and using other treatments nutritional and pharmacological values can be enhanced. 

Micronutrients are vital for plant growth because they act as a cofactor of the enzyme, take part in redox reactions, and have several other important functions [14]. Furthermore, despite addition to the soil, micronutrient application using seeds improves the stand formation, advances phenological events, and increases yield and micronutrient grain contents [14]. Like different micronutrients, molybdenum (Mo) is very vital and essential for plants’ physiological functions. In plants with inadequate Mo, nitrates accrue in leaves, which then do not assimilate into proteins. In legumes, Mo serves an additional function: to help root nodule bacteria to fix atmospheric nitrogen (N). Studies revealed that the application of Mo in beans, via seeds, increases the mass of the nodule [15]; moreover, its foliar spray increases nodule formation, nitrogen contents, the grain number, the grain mass, and overall productivity [16]. The enhanced benefits may be attributed to the acceleration of N absorption and assimilation processes via BNF (biological nitrogen fixation) [14]. Mo is a crucial element for more than 40 enzymes, four of which have been found in plants, including nitrate reductase (NR) and nitrogenase synthesis [17,18]. Thus, plants receiving increased Mo will have increased N productivity levels based on the higher activities of the abovementioned enzymes. Mo bound to a unique pterin compound, named Mo cofactor, binds to diverse apoproteins and aids in anchoring the Mo center at the correct position within the holo-enzyme for its correct interaction with other components of the electron transport chain [17]. Thus, Mo supply can strengthen plant metabolism at different growth stages through an improved enzymatic and non-enzymatic antioxidant defense system and also enhance other pharmacological properties [19]. Moreover, Mo is also an essential mineral for the human body as it is part of important metabolic enzymes such as xanthine oxidase sulfite oxidase, aldehyde oxidase, and mitochondrial amidoxime reducing component.

With this background, we hypothesized that Mo application can enhance the nutritional and pharmacological value of *Canavalia* species by improving their N content and resulting in primary and secondary metabolite production. Thus, the present study aimed to evaluate the impact of Mo seed priming on three *Canavalia* species/cultivar sprouts. To the best of our knowledge, no study has been conducted to assess the influence of Mo seed priming on *Canavalia ensiformis var. gladiata*, *Canavalia ensiformis var. truncata Ricker*, and *Canavalia gladiata var. alba* Hisauc. Herein, we evaluated the impacts of Mo treatment on growth, N content, and phenolic metabolism as well as on the concentrations of several phytochemical compounds. We further examined the role of Mo in the enhancement of pharmacological properties of *Canavalia* species. Overall, our study contributed to an understanding of the biochemical basis of Mo that induces high growth and tissue quality of different *Canavalia* species/cultivars.

## 2. Results

### 2.1. Biomass and Pigment Content

**Figure 1:**
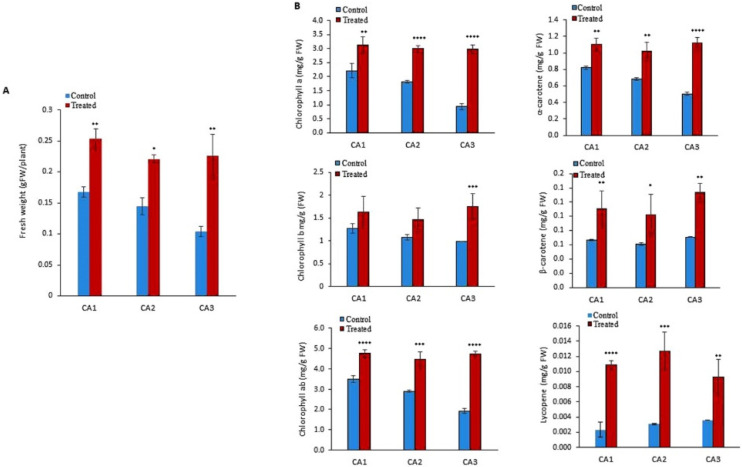
Impacts of molybdenum (Mo) seed priming on different *Canavalia* species. (**A**): Total biomass (gFW). (**B**): Chlorophyll a, b, and ab content, α-carotene content, β-carotene content, and lycopene content were measured and expressed as mg/gFW. Control; without treatment, Treated; Mo treatment. CA1; *Canavalia ensiformis var. gladiata*, CA2; *Canavalia ensiformis var. truncata Ricker*, CA3; *Canavalia gladiata var. alba Hisauc*. α-carotene, *or* β-carotene, Beta-carotene. The bars above mean indicate ± standard deviation (S.D) of three independent replicates (*n* = 3). Asterisks (*) show the level of significance * *p* < 0.05, ** *p* < 0.01, *** *p* < 0.001, **** *p* < 0.0001.

To quantify the biomass of the molybdenum (Mo)-treated cultivar/species of *Canavalia*, the validated method was employed. An increase in fresh weight was observed in all the sproutings of the three studied species/cultivars, as shown in Figure 1A. Significant differences were observed among Mo-treated and non-treated groups. The difference was almost negligible within the sproutings of different species. Next, different types of chlorophylls such as chlorophyll a, chlorophyll b, and chlorophyll ab were quantified in both Mo-treated and untreated plants. The results indicated that exogenous Mo application increased chlorophyll content in the leaves of all the studied *Canavalia* species, as shown in Figure 1B. In untreated sprouts of *Canavalia ensiformis var. gladiata* (CA1), *Canavalia ensiformis var. truncata* *Ricker* (CA2), and *Canavalia gladiata var. alba Hisauc* (CA3), chlorophyll ab was present in a higher amount as compared to chlorophyll a and b, which was significantly increased after seed priming with Mo, as shown in Figure 1B. Further, the impact of Mo on the production of carotenoids was studied. Concentrations of α-carotene, β- carotene, and lycopene were quantified. A significant increase in the production of T-carotene and B-carotene was observed in all the Mo-treated species/cultivars, while the concentration of lycopene remained the same, as shown in Figure 1B.

### 2.2. Nutritional Value 

The variations in the concentration of nutrients (lipids, proteins, and fibers) and phytochemicals (alkaloids, flavonoids, saponins, and glycosides) were quantified in both Mo-exposed and unexposed seeds of *Canavalia*. The quantity of total lipids was significantly increased in CA1, a non-significant change was observed in CA2, while a decrease was recorded in CA3. Furthermore, a significant increase in fiber content was recorded in CA1, a decrease was found in CA3, while there was no change in the fiber content of CA2. Upward trends in total proteins, flavonoids, saponins, and glycosides were noticed (Table 1). 

### 2.3. Nitrogen and Amino Acid Metabolism 

**Figure 2:**
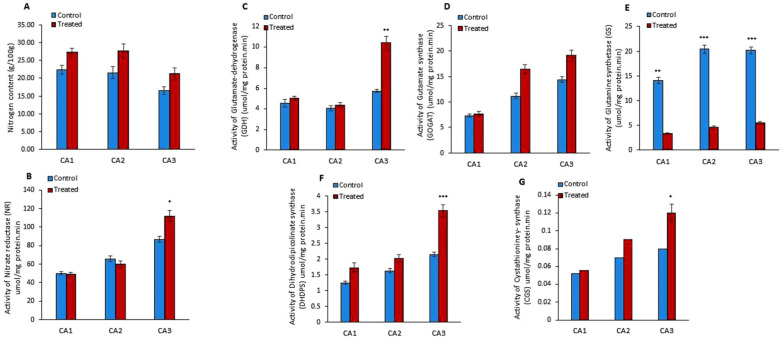
Effects of molybdenum (Mo) treatment on nitrogen assimilation. (**A**): Nitrogen (N) content (g/100gFW). (**B**): Nitrate reductase (umol/mg protein. min) activity. (**C**): Glutamte dehydrogenase (GDH) activity (µmol/mg protein. min). (**D**): Gutamate synthase (GOGAT) activity (µmol/mg protein. min). (**E**): Glutamine synthetase (GS) activity (µmol/mg protein. min). (**F**): Dihydrodipicolinate synthase (DHDPS) activity (µmol/mg protein. min). (**G**): Cystathionine γ- synthase (CGS) activity (µmol/mg protein. min). Control; without treatment, Treated; Mo treatment. CA1; *Canavalia ensiformis var. gladiata*, CA2; *Canavalia ensiformis var. truncata Ricker*, CA3; *Canavalia gladiata var. alba Hisauc*. Experiments were carried out in triplicate and the data are expressed as the mean ± standard deviation; Level of significance * *p* < 0.05, ** *p* < 0.01, *** *p* < 0.001.

The outcome of Mo treatment on the nitrogen (N) content was studied in *Canavalia* species. The results showed a slight increase in N production in the sproutings of three species, as shown in Figure 2A. Moreover, the quantification of nitrogen reductase (NR) activity showed its increase in CA3 and decrease in CA1 and CA2 in Mo-treated plants, as shown in Figure 2B. Next, we studied the effect of Mo on two pathways of glutamate synthesis, i.e., glutamate-dehydrogenase (GDH) and glutamine synthetase (GS)/glutamate synthase (GOGAT) pathways. The activities of GDH, GOGAT, and GS were measured (Figure 2C–E). The results showed an increase in GDH and GOGAT activity in the three species. The rise in GDH activity was significant in CA3, while it was non-significant in other species. Inversely, GS activity was decreased after Mo treatment and the differences were highly significant in the three species/cultivars. Further, the impact of Mo treatment on dihydrodipicolinate synthase (DHDPS) and cystathionine γ- synthase (CGS) activity was explored. The experiment showed that the activity of DHDPS significantly increased in CA3 and non-significantly in CA1 and CA2 (Figure 2F). Moreover, a notable increase in the activity of CGS was measured in sprouts of CA3 (Figure 2G). Furthermore, amino acids, i.e., asparagine, glutamine, glycine, glutamic acid, isoleucine, arginine, tyrosine, lysine, serine, alanine, proline, histidine, leucine, isoleucine, valine, cystine, threonine, tryptophan, and methionine, were quantified in Mo-treated *Canavalia* species and data were compared to untreated controls. The changes in each amino acid content due to Mo exposure are shown in Table 2. Overall, an increase in amino acid production was detected in the three species. The quantity of cystine, isoleucine, leucine, glycine, alanine, and proline was increased significantly in CA1; asparagine, glutamine, glutamic acid, alanine, proline, cystine, and lysine were enhanced in CA2; and asparagine, glutamine, glycine, histidine, methionine, cystine, isoleucine, tyrosine, lysine, threonine, and tryptophan were enhanced in CA3. Variable trends were noticed among different sprouts. The concentrations of alanine and proline were found to be improved in CA1 and CA2, while they declined in CA3. Levels of histidine, arginine, valine, threonine, and tryptophan increased in CA1 and CA3, whereas they decreased in CA2. Leucine was increased in CA1 and reduced in CA3 and CA2. All results are enumerated in Table 2. 

### 2.4. Impact of Mo Treatment on Phenolic Level and Metabolism 

#### 2.4.1. Total Phenolic Content

The impact of Mo on the total phenolic content of *Canavalia* species was evaluated. The total phenolic content was expressed as gallic acid equivalent (GAE). The results indicated that the production of phenols was increased significantly in CA3. A non-significant increase was observed in CA2, whereas a decrease was noticed in CA1, as given in Table 1. 

#### 2.4.2. Phenolic Compounds

Further, we evaluated the quantity of individual phenolic compounds in the Mo-treated group and compared them with untreated controls. Different phenols, i.e., gallic acid, caffeic acid, *p*-coumaric acid, chicoric acid, rosmarinic acid, protocatechuic acid, quercetin, naringenin, kaempferol, luteolin, apigenin, naringenin, rutin, and chlorogenic acid, were quantified using HPLC. The results revealed that Mo impacted the synthesis of different phenols in an interesting pattern among different species of *Canavalia*. Gallic acid, *p*-coumaric acid, rosmarinic acid, naringenin, and chlorogenic acid were enhanced significantly in CA1; caffeic acid, *p*-coumaric acid, chicoric acid, rosmarinic acid, naringenin, and apigenin were increased significantly in CA2; and gallic acid, chicoric acid, kaempferol, and chlorogenic acid were raised significantly in CA3. Enhanced production of caffeic acid, rosmarinic acid, and luteolin was observed in all three species after exposure to Mo. Interestingly, differential patterns were observed for other phenols (Table 3). Gallic and chlorogenic acid production was induced by Mo in CA1, while a reduction pattern was observed in CA2 and CA3. On the other hand, the biosynthesis of chicoric acid, protocatechuic acid, quercetin, and naringenin was decreased in CA1 and increased in CA2 and CA3. Moreover, *p*-coumaric acid and kaempferol were raised in CA1 and CA2 and reduced in CA3. Interestingly, apigenin synthesis was not affected by Mo treatment in CA2, while a reduction was found in CA2 and CA3—values are given in Table 3.

#### 2.4.3. Phenolic Metabolism

**Figure 3:**
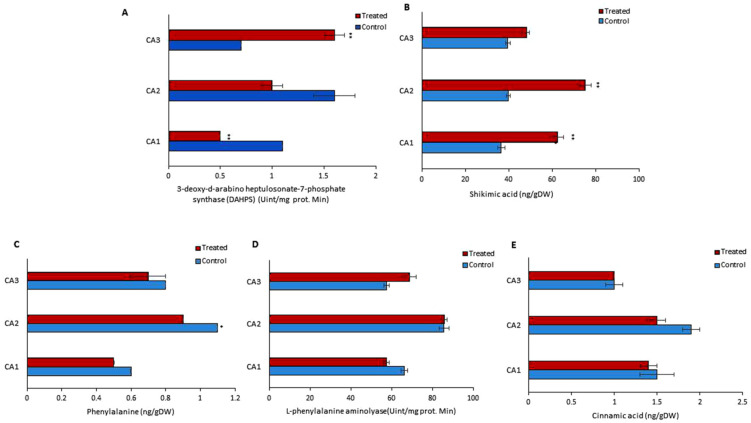
Effects of molybdenum (Mo) treatment on phenol synthesis pathway. (**A**): 3-deoxy-d-arabino heptulosonate-7-phosphate synthase (DAHPS) activity. (**B**): Shikimic acid level. (**C**): Phenylalanine. (**D**): L-phenylalanine aminolyase activity. (**E**): Cinnamic acid content. Control; without treatment, Treated; Mo treatment. CA1; *Canavalia ensiformis var. ladiate*, CA2; *Canavalia ensiformis var. ladiate Ricker*, CA3; *Canavalia ladiate var. alba Hisauc*. The bars above mean indicate ± standard deviation (S.D) of three independent replicates (*n* = 3). Asterisks (*) show the level of significance, * *p* < 0.05, ** *p* < 0.01.

Various parameters were determined to assess the endogenous metabolism of phenols in treated and untreated groups. For this purpose, the concentrations of phenylalanine, L-phenylalanine aminolyase, cinnamic acid, 3-deoxy-d-arabino heptulosonate-7-phosphate synthase (DAHPS), and shikimic acid were quantified (Figure 3). An inverse relation of DAHPS and shikimic acid was observed in the treated group of CA1 and CA2 (Figure 3A,B). The DAHPS activity was increased significantly in CA3, while it was decreased in CA1. A significant rise in the concentration of shikimic acid was observed in both CA2 and CA1. Phenylalanine was reduced in CA1, CA2, and CA3, but the change was not significant (Figure 3C). In the case of L-phenylalanine aminolyase, we found that its activity was increased in CA2 and CA3, though decreased in CA1, after the application of Mo (Figure 3D). Cinnamic acid was decreased in CA1 and CA2, but in the third species an upward trend was observed upon Mo exposure. Cinnamic acid metabolism was not affected in CA3, while a decreasing pattern was found in CA1 and CA2 (Figure 3D).

### 2.5. Impact of Mo Treatment on Biological Activity

**Figure 4:**
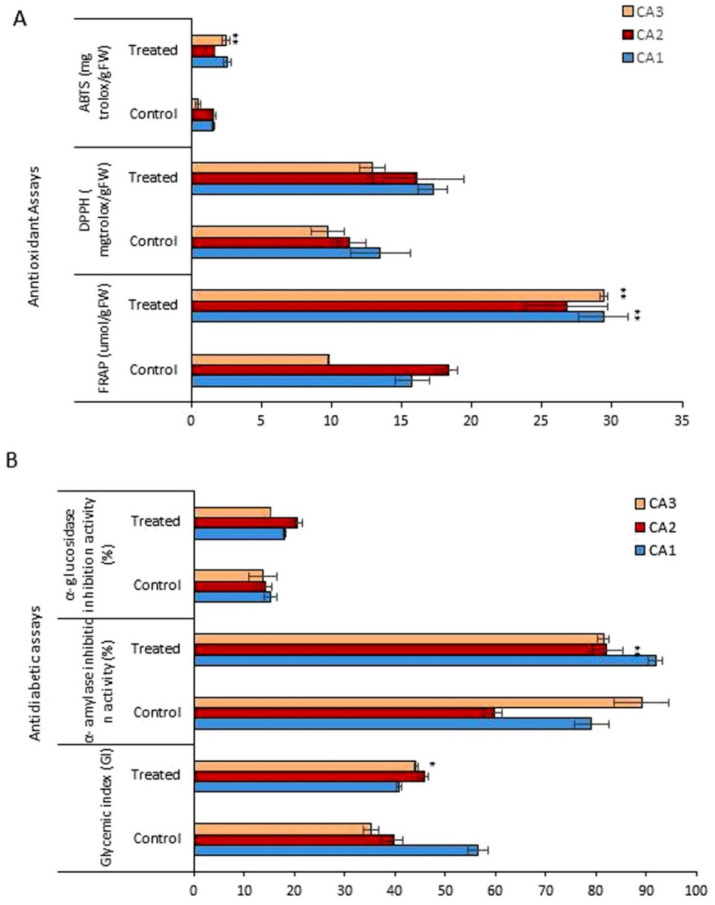
Effects of molybdenum (Mo) treatment on antioxidant and antidiabetic properties of *Canavalia* species (**A**): Antioxidant property, i.e., FRAP, ABTS, and DPPH. (**B**): Glycemic index (GI), α-amylase inhibition activity, and α-glucosidase inhibition activity. Control; without treatment, Treated; Mo treatment. CA; *Canavalia ensiformis var. gladiata*, CA2; *Canavalia ensiformis var. truncata Ricker*, CA3; *Canavalia gladiata var. alba*. The bars above means indicate ± standard deviation (S.D) of three independent replicates (*n* = 3). Asterisks (*) show the level of significance according to analysis of variance (ANOVA), * *p* < 0.05, ** *p* < 0.01.

Figure 4 shows the antioxidant activity, quantified by the 2,2-diphenyl-1-picryl-hydrazyl-hydrate (DPPH) free radical scavenging, 2,2-azino-bis(3-ethylbenzothiazoline-6-sulfonic acid) (ABTS), ferric reducing antioxidant power (FRAP) methods, for the three plant species/cultivars of *Canavalia* after Mo exposure. The antioxidant activity, measured for each of the species, corresponds to an extract and Mo concentration. Regarding the antioxidant activity quantified by ABTS, the three species exhibited different antioxidant potentials, which was further increased after Mo exposure (Figure 4A). The antioxidant capacity pattern in controls was observed as CA1 > CA2 > CA3. Mo treatment induced a significant increase in CA3. Further, the values obtained from the FRAP assay also revealed the dramatic increase in antioxidant activity after Mo exposure, and a significant increase was recorded in both CA1 and CA3. Next, for the antioxidant activity quantified by DPPH, an increasing trend was obtained in all three species in the Mo-treated group, but the difference was non-significant. The glycemic index (GI), α-amylase inhibition activity, and α-glucosidase inhibition activity were assessed to explore the impact of Mo on the antidiabetic potential of *Canavalia* species. Through GI, a significant increase in antidiabetic potential was recorded in CA3, but in CA1 a decrease was noted. Further, the α-amylase and α-glucosidase inhibition potentials of *Canavalia* species were also increased. A-amylase inhibition was highly significant by CA2. A non-significant decrease was observed in CA3, as shown in Figure 4B. The evaluated percentage values of the inhibition of α-glucosidase revealed that Mo positively enhanced the inhibition potential of the three species/cultivars of *Canavalia*.

### 2.6. Principle Component Analysis (PCA) 

**Figure 5:**
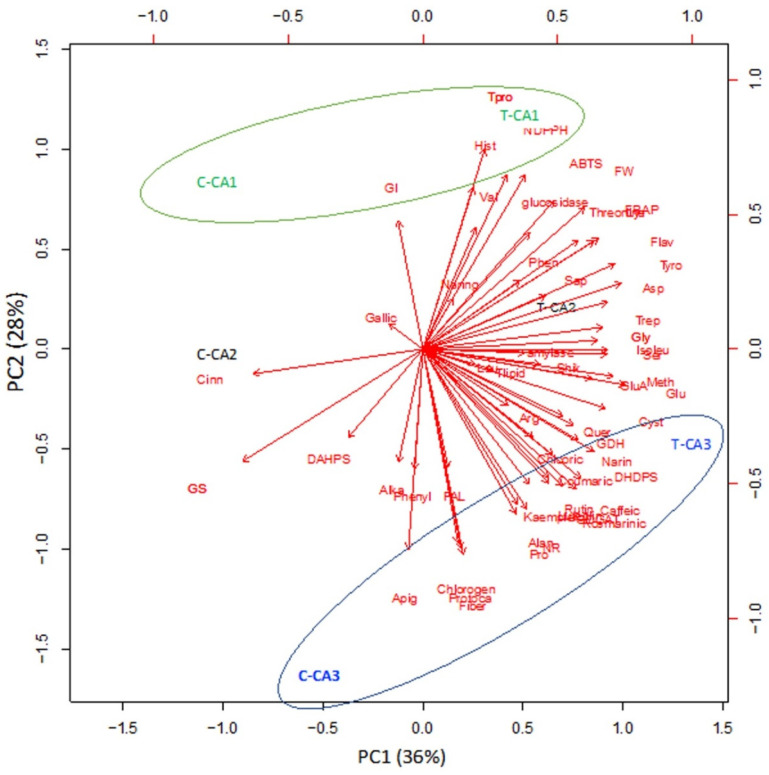
Principle component analysis (PCA) of 3 *Canavalia* species after seed priming with molybdenum (Mo). CA; *Canavalia ensiformis var. gladiata*, CA2; *Canavalia ensiformis var. truncata Ricker*, CA3; *Canavalia gladiata var. alba Hisauc*.

R software was used to perform principal component analysis (PCA). For this, Mo-treated and untreated species of *Canavalia* were chosen to analyze the interrelationship of amino acid content, phenolics, antioxidant activities, and antidiabetic activities. Here, the 1st principal component (PC1) showed 36% of the variance, while the 2nd principal component showed 28% variance between untreated and Mo-treated *Canavalia* species, as shown in Figure 5. PC1 vs. PC2 showed significant differences among Mo-treated and untreated plant species as well as between CA1 and CA3. Both PCs displayed positive correlations among most of the parameters. PC1 was highly and positively correlated with many phenols, such as rutin, caffeic acid, kaempferol, rosmarinic acid, naringenin, and coumaric acid, etc., and amino acids including alanine, proline, cystine, and methionine, etc., whereas PC2 was positively related to histidine and valine. The results showed that all phenolic acids, as well as the total amount of phenols, differed with Mo treatment, whereas the treatments and species of *Canavalia* were well separated in the map of PCA. Moreover, the results of antioxidant activities also showed a positive correlation, assessed by ABTS and FRAP assays. 

## 3. Discussion

The present study was conducted to explore the effects of seed priming using Mo on different endogenous chemical parameters of three species/cultivars of *Canavalia*. For this study, 0.1% ammonium molybdate was used and effects were monitored in sproutings of *Canavalia ensiformis var. gladiata* (CA1), *Canavalia ensiformis var. truncata Ricker* (CA2), and *Canavalia gladiata var. alba Hisauc* (CA3). We used the seed priming method as several studies indicated that seed treatment is a more efficient method for Mo than soil application [20,21]. Kumar Rao et al. reported that seed priming with Mo (0.5 g L^−1^ solution of sodium molybdite) increased the yield to 27%, compared to Mo soil application, in a pot study on chickpea [22]. However, genetic variations led to interesting differential patterns among different species/cultivars in our study, which we have discussed. Similarly, the results also revealed that Mo has not targeted the biosynthetic pathways uniformly. 

### 3.1. Increased Canavalia Biomass Production by Improving Photosynthetic Pigments 

Micronutrients are crucial for plant growth and development. Their deficiency contributes to a reduction in growth and changes in photosynthesis due to variations in pigment synthesis. Increased chloroplast deformation, the over-production of antioxidant enzymes, and increased production of proteins are the most common signs of stress-related responses in plants. Mo, a micronutrient, acts as a cofactor for several enzymes, thus helping to promote plant growth and biomass [23], and its exposure can lead to dramatic effects on *Canavalia* species. In the current study, the biomass of sproutings, in both Mo-treated and untreated groups, was quantified. Our findings revealed that Mo treatment significantly increased the fresh weight of sproutings in all the studied *Canavalia* species/cultivars. Alam et al. also observed that Mo application proportionately enhanced the weight of nodules in hairy vetch roots [24]. This observation might be attributed to the fact that plants require micronutrients for biosynthetic pathways and plant growth [25]. 

Next, the impact of Mo on the photosynthesis process was assessed, for which pigments such as chlorophyll and carotenoids play an important role. Chlorophyll, which includes chlorophyll a, chlorophyll b, and chlorophyll ab, is a green pigment for photosynthesis. Carotenoids are also important pigments that exhibit antioxidant and provitamin A roles and are used extensively as natural and safe colorants for food and cosmetics [26]. In our study, exogenous Mo increased chlorophyll content in *Canavalia*. Indeed, Mo stabilizes the structure of chloroplasts, enhances the volume and number of chloroplast grana, and increases the synthesis of chlorophyll [27]. In agreement with the current results, it has been demonstrated that micronutrient Mo application enhanced chlorophyll content in Mung Bean (*Vigna radiata* L.) when its seeds were primed with Mo [28]. 

### 3.2. Improved Nitrogen (N) Content and Amino Acid Metabolism

Amino acids are the main part of the cellular structure. Their synthesis costs in terms of energy are expected to play an important role in energy allocation. It was experimentally shown that the source of nitrogen, predominantly nitrate and/or ammonium, affects amino acid and protein levels, as well as the rate of growth and, consequently, overall biomass [29]. Nitrogen assimilation by plants directly takes part in the synthesis and conversion of amino acids through the reduction of nitrate. In our study, Mo application improved N metabolism and as a result increased total protein content and amino acids in the *Canavalia* sprouts. Similar to our results, the study of Toledo et al. showed that Mo foliar application increased nitrogenase and nitrate reductase activities, which resulted in an increase in N accumulated in the soybean leaves [30]. Among the molybdo-enzymes, nitrate reductase (NR) represents the cytosolic key enzyme of nitrogen assimilation that reduces nitrate to nitrite [31]. We have observed that the Mo priming of seeds significantly increased NR expression in CA3, which is in line with the study of Camp et al. In that study, Mo treatment resulted in a rise in total grain N in soybean seeds [32]. Interestingly, this increase was not observed in the sprouting of CA1 and CA2, which might be due to different genetic backgrounds. Mechanistically, N production via Mo priming led to the modulation of many biochemical reactions of *Canavalia* species, which was observed in different experiments of the study. Normally, all N taken up by plants is first reduced to NH4+ because it is the only reduced N form available to plants for assimilation into N-carrying amino acids [33]. Ammonium is then integrated into glutamine and glutamate. It was discovered that there are two pathways for glutamate synthesis. The glutamine synthetase (GS)/glutamate synthase (GOGAT) pathway is believed to assimilate ammonia at normal intracellular concentrations, while GDH (glutamate-dehydrogenase) plays a key role in the assimilation of ammonia into amino acids [34]. In our study, an increase in GDH and GOGAT production was detected in the three species, which was significant in CA3. GS production decreased in sprouts of the treated group and the change was significant in all three species/cultivars. The results are in line with the study of Imran et al., where Mo application up-regulated the expressions of GOGAT genes in winter wheat (*Triticum aestivum* L.) under a sole NH_4_^+^ source [35,36]. Moreover, dihydrodipicolinate synthase (DHDPS) and cystathionine γ-synthase (CGS) are the first committed step in the biosynthesis of lysine and methionine, respectively, which occurs naturally in plants [37,38]. We observed an increase in the activity of DHDPS and CGS, which consequently increased the production of lysine and methionine in Mo-treated *Canavalia* species. The increase was significant in CA3, which might be due to the more prominent NR activity in CA3. 

The amino acids in plants are not only essential components of protein synthesis, but also serve as precursors for a wide range of secondary metabolites that are important for plant growth as well as for human nutrition and health. The enhanced quantity of most of the amino acids in the study evidenced that there was an additional availability of amino acids, probably due to the greater biological fixation of N enhanced by Mo for the enzyme nitrogenase and the production of a greater quantity of ureides. Ureides are transported from the nodules to the aerial part and later converted into amino acids. The process resulted in a greater source of amino acids and, consequently, a higher protein content, as observed in our study. Conversely, Toledo et al. did not observe an increased protein content with applications of 30 and 60 g ha^−1^ Mo applied by leaf spray or with 24 g ha^−1^ per seed treatment [30], which might be due to differences in species and/or different underlying biosynthetic mechanisms. Similar to our study, Oliveira et al. found a linear increase in the protein content of soybean when they applied doses of 0 to 800 g ha^−1^ Mo via foliar application [39]. In the current study, most of the amino acids were significantly increased in CA3 as compared to CA1 and CA2. This increase correlates to enhanced N metabolism and a significant increase in the biomass of CA3.

### 3.3. Improved Nutritional Value 

Furthermore, the quantification of nutrients in sprouts showed a substantial increase in the lipid content of CA1, which might be due to increased synthesis and/or decreased degradation of lipids. Previous documents show that increased photosynthetic capacity due to Mo supply contributes to the accumulation of carbohydrates, such as fructose and glucose. We observed that lipids, fibers, and saponins were significantly increased in CA1. Flavonoids were increased reasonably in CA3. No significant change was observed in CA1 in all the studied parameters. Different impacts of Mo were observed in different species/cultivars, which might be due to different genetic backgrounds and the variable uptake of Mo. 

### 3.4. Secondary Phenolic Production Improvement as a Basis for High Bioactivity 

Phenolics play essential roles in plant development; these aromatic benzene ring compounds with one or more hydroxyl groups are produced by plants mainly for protection against stress [40]. In our study, the Mo priming of *Canavalia* seeds significantly increased the phenolic content of CA3 sprouts; a non-significant increase was found in CA2 and, surprisingly, a slight decrease was noticed in CA1. This might be due to differences in endogenous molecular pathways of different species. This link to the inherent levels of mechanisms may also lead to differences in the enhancing patterns of the individual phenolic compounds after Mo treatment, which we observed in our study. A similar trend was observed in DAHPS, the first enzyme of the shikimate pathway, which converts PEP and E4-P into 3-dehydroquaianate. Further, to assess the impact of Mo seed priming and enhanced phenolic production on the pharmacological properties of plants, we performed antioxidant and antidiabetic assays. FRAP, ABTS, and DPPH assays were used to evaluate the antioxidant capacity of species samples spectrophotometrically. An increase in antioxidant potential was observed after Mo treatment in all species. In the FRAP assay, the increase was significant in both CA1 and CA3. CA3 also expressed a significant change in antioxidant activity, measured via ABTS assay. This correlates to the above findings that more impacts of Mo on synthetic pathways were observed in CA3. Antioxidant activity has recently become a target for product development in the pharmaceutical and cosmetics industries [41]. *Canavalia* species are of medicinal importance due to their potential antioxidant properties. In a study, *Canavalia gladiata* extract, at the concentration of 2 mg/mL, showed an antioxidant effect comparable to that of ascorbic acid of the control group [42]. Plant growth and antioxidant capacity are greatly dependent upon N availability. Higher N improves the stress tolerance of plants via enhancement of the antioxidant ability and inhibition of lipid peroxidation [43]. Mo primarily improves the nitrogen fixation to the plant and increases its antioxidant potential, which we observed in our study. This also might be the reason for the enhanced antidiabetic potential of *Canavalia* species assessed by GI, α-amylase inhibition activity, and α-glucosidase inhibition activity. Terpenoids and flavonoids of *Canavalia gladiata* are reported to play a role in lowering glucose levels and possessing antioxidant potential [44]. The Mo-mediated enhanced concentrations of terpenoids and flavonoids might have led to the increased antioxidant and antidiabetic activities of *Canavalia* sprouts in our study.

## 4. Materials and Methods

### 4.1. Plant Material and Growth Conditions

**Figure 6:**
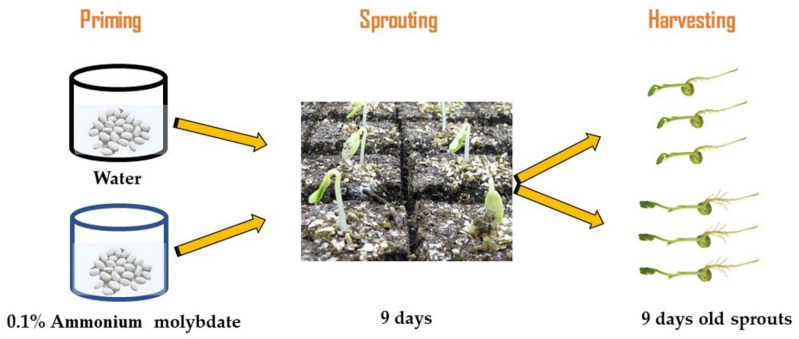
Flow chart of the experimental design.

Seeds of three *Canavalia species*: *Canavalia ensiformis var. gladiata* (CA1), *Canavalia ensiformis var. truncata Ricker* (CA2), and *Canavalia gladiata var*. *alba Hisauc* (CA3), were collected from the Agricultural Research Center (Giza, Egypt). Healthy seeds with a similar shape and size were washed with distilled water and dripped for 1 h in 5 gL^−1^ sodium hypochlorite, and then they were washed thoroughly with distilled water. According to a preliminary experiment, regarding the effect of ammonium molybdate (Mo) priming for 10 h at six concentrations, 0 (distilled water) and 0.025, 0.05, 0.075, 0.1, and 1.5% ammonium molybdate, 0.1% Mo-treated plants showed the highest biomass accumulation, thus 0.1% was selected to study the effect of Mo-priming on sprouts and tissue chemical composition of *Canavalia species*. About 250 seeds were primed for 10 h with distilled water or Mo (0.1%) at room temperature (24 °C). For sprouting processes, the seeds were distributed on trays filled with vermiculite and irrigated with Milli-Q water every two days. Then, the seeds were evenly transferred to trays covered with vermiculite and moistened with 150 mL of aquaponic water (mineral-rich water that was collected from a catfish tank and adjusted to a target pH of 7). The growth conditions were 150 μmol PAR m^−2^ s^−1^, 23/18.5 °C air temperature, 63% humidity, and 16/8 h day/night photoperiod. The sprout tissues from each treatment were harvested after 9 days and weighed (Figure 6), then they were frozen in liquid nitrogen and kept at −80 °C for biochemical analyses. Each experiment was replicated at least two times, and for all assays, 3 to 5 replicates were used, and each replicate corresponded to a group of sprouts and mature plants harvested from a certain tray.

### 4.2. Preparation of Extracts

For sample preparation, we used an ETA 0067 grinder with grinding stones, VIPO mini grinder, followed by homogenization by Vibrom S2 (Jebavý, Trebechovice p. O., Czech Republic), and a cryogenic grinder accompanied by liquid nitrogen. Supercritical fluid extraction (SFE) using SE-1 (SeKo-K, Brno, Czech Republic) extractor and a steam distillatory apparatus according to CSN 58 0110 and CSN 6571 were successively applied for extraction and subsequent determination of the total content of *Canavalia* oils. Approximately 500 mg of each sample was transferred into an extraction column for SFE extraction. The extraction was performed at 40 MPa for 60 min, and extractor and restrictor temperatures were 80 and 120 °C, respectively. The extract was further trapped into a hexane layer inside a trapping vessel.

### 4.3. Pigment Analysis

To extract pigments, samples were homogenized in acetone and then centrifuged (4 °C, 14,000× *g*, 20 min). HPLC (Shimadzu SIL10-ADvp, Japan, reversed-phase, at 4 °C) was used to analyze the obtained solution (Almuhayawi et al., 2020). Carotenoids were separated (silica-based C18 column, Waters Spherisorb, 5 μm ODS1, 4.6 × 250 mm), whereas they were eluted by (A) acetonitrile: methanol: water (81:9:10) and (B) methanol: ethyl acetate (68:32). The pigment was quantified using a diode-array detector (Shimadzu SPDM10Avp, Tokyo, Japan) at four wavelengths (420, 440, 462, and 660 nm). Pigments were identified by comparing the standard mixture to the relative retention time of each pigment and the concentrations were calculated using the peak area of the corresponding standard.

### 4.4. Determination of Nitrogen Content and Metabolism

For total nitrogen (N) content, fine ground sprout samples (0.2 g) were digested in H_2_SO4–H_2_O at 260 °C, and the nitrogen levels were assessed with a CN element analyzer (NC-2100, Carlo Erba Instruments, Milan, Italy). Glutamine synthetase (GS, EC 6.3.1.2), glutamate synthase (EC 1.4.7.1), and glutamine 2-oxoglutarate aminotransferase (GOGAT, EC 1.4.7.1) enzyme activities were measured by monitoring the reduction of NADH at A_340_. GDH determining 2-oxoglutarate-dependent NADH oxidation. GS activity was detected by assessing the formation of γ-glutamyl hydroxamate at A340. GOGAT activity was estimated by following the glutamine-dependent NADH oxidation at A340. The activity of the nitrate reductase (NR, EC 1.7.1.1) enzyme was measured by following the nitrite-dependent NADH oxidation (A_340_) [45]. Protein concentrations were determined according to Lowry et al. [46].

### 4.5. Amino Acid Analysis 

About 3 mg of each *Canavalia* sample was hydrolyzed with 6 M HCl (6 h, 150 °C), and the acidic suspension was evaporated by rotary evaporation (RE500 Yamato Scientific America Inc., Santa Clara, CA, USA) and redissolved in 2 mL of sodium citrate buffer (pH 2.2). For the derivation step, phthalaldehyde (OPA) (7.5 mM) was mixed with samples in citrate buffer (OPA reagent containing β-mercaptoethanol and Brij 35). The HPLC (Shimadzu SPDM10Avp, Tokyo, Japan) analysis was evaluated using internal and external standards with the aid of fifteen amino acid reference standards (0.05 µmoles mL^−1^ amino acid) for retention time detection of each single amino acid. In the meantime, the internal standard (0.05 µmoles mL^−1^ α aminobutyric) was added to both the plant sample and the reference. Reversed-phase C18 column (100 × 4.6 mm × 1/4” Microsorb 100-3 C18, Agilent Technologies, Santa Clara, CA, USA) was used and gradient elution was performed by mobile phase consisting of 0.1 M sodium acetate and methanol (9:1). Measurement was at a wavelength of 360 and 455 nm. Star Chromatography software (Varian version 5.51) was applied for amino acid peak integration and final calculation was carried out to express values as µmol/gFW.

### 4.6. Determination of Total Carbohydrates, Protein, Lipids, and Fibers

Carbohydrate evaluation was processed for each *Canavalia* sample (eCO_2_-treated and control plants) by Nelson’s method [47], while the concentration of protein was measured for each frozen *Canavalia* sample (0.2 g FW). Detection of total lipids was performed using Folch’s method [48], whereas the samples were subjected to homogenization in a chloroform/methanol mixture (2:1), followed by centrifugation at 3000× *g* for 15 min and concentration of chloroform extract containing lipids via a rotary evaporator (RE500 Yamato Scientific America Inc.); after that, the produced pellets were re-dissolved in a mixture of toluene/ethanol (4/1 *v*/*v*) and then mixed with saline solution, re-concentrated again, and weighed to calculate the total lipid content (µg/gFW). Fibers were also extracted and evaluated according to the AOAC method [49], starting with sample gelatinization using α-amylase (30 min, pH 6, 100 °C), then enzymatic digestion by protease (30 min, pH 7.5, 60 °C). Thereafter, starch and proteins were removed by amyloglucosidase (30 min, pH 6 and 0 °C). Finally, fibers were precipitated using ethanol, washed, and the yielded residue was weighed and expressed as µg/gFW.

### 4.7. Determination of Phenolics and Their Precursors and Related Enzymes

The determination of phenolics and their precursor metabolites was carried out using an ultra-performance liquid chromatography system (Waters Acquity UPLC, Boston, MA, USA) coupled with a quadrupole mass spectrometer (Waters Xevo TQ, Manchester, UK) provided with an ESI source according to Wang et al.’s method [50]. Phenolic and flavonoid levels were identified by comparing the standard mixture of different phenols and flavonoids to the relative retention time. The concentration of each compound (µmol/gFW) was calculated using the peak area of the corresponding standard.

In addition, deoxy-d-arabino 201 heptulosonate-7-phosphate synthase (DAHPS) activity (umol/mg protein. min) was analyzed according to Wang et al., (2020). This enzyme catalyzes the reactions in cinnamic and shikimic acid pathways that are involved in phenylpropanoid biogenesis and therefore in the biosynthesis of coumarins. Samples were first homogenized in 3 mL 50 mM Tris-HCL buffer (pH = 7.5). The assay mixture contained 0.1 mM erythrose-4-phosphate, 0.2 mM phosphoenolpyruvate, and 0.1 mM MnSO_4_/0.1 mM CoCl2. In total, the reaction was initiated by enzyme addition and terminated by 25% (*w*/*v*) trichloroacetic acid addition. The activity of DAHPS was measured at 549 nm. Regarding PAL, it was extracted in 1 mL of 200 mM sodium borate buffer (pH 8.8) and then evaluated by measuring the production of trans-cinnamic acid at 290 nm. 

### 4.8. Measurement of Antioxidant Capacity 

The determination of antioxidant activity was performed through ferric reducing antioxidant potential, 2,2-diphenyl-1-picryl-hydrazyl-hydrate (DPPH) free radical scavenging, 2,2-azino-bis(3-ethylbenzothiazoline-6-sulfonic acid) (ABTS), and ferric reducing antioxidant power (FRAP) assays according to the reported method [51]. In the FRAP method, about 0.2 g of *Canavalia* samples was firstly extracted in ethanol (80%) and centrifuged at 14,000 rpm for 20 min. Afterward, 0.1 mL of tested extracts was added to 0.25 mL of FRAP reagent (20 mM FeCl_3_ in 0.25 M acetate buffer, pH 3.6) at room temperature, the absorbance was further measured at 517 nm, and antioxidant activity is expressed as µmol/ gFW. In the DPPH assay, the reaction mixture was composed of 3.9 mL of 200 μM DPPH (prepared in ethanol) and 0.1 mL of samples, incubated in the dark for half an hour at room temperature (35 ± 2 °C), and the absorbance was further detected at 517 nm. The percentage of inhibition was calculated versus a control. ABTS (2,2′ -azino-bis(3-ethylbenzothiazoline-6- sulfonic acid) scavenging activity was measured by mixing ABTS with potassium persulphate (2.4 mM), then the reaction was performed for 12 in the dark, and absorbance was measured at 734 nm and antioxidant activity was calculated as trolox/ gFW.

### 4.9. Anti-Diabetic Activity 

#### 4.9.1. Determination of In Vitro Glycemic Index (GI) 

In vitro starch hydrolysis assays were used for the evaluation of GI [52]. Sprouts were powdered and incubated with pepsin (100 mg/mL) in a reaction buffer (HCl-KCl buffer, pH 1.5), incubated for 1 h at 40 °C with shaking, and then the mixture was diluted in phosphate buffer (pH 6.9), adding α-amylase and incubation at 37 °C. Approximately 1mL of aliquots was taken every 30 min and boiled for 20 min for the sake of amylase enzyme inactivation. Residual starch was digested to glucose by adding 60 μL amyloglucosidase together with 0.4 M of sodium acetate buffer (pH 4.75), and the reaction mixture was previously incubated at 60 °C for 50 min. Aliquots (0.6-mL) were incubated with 1.2 mL glucose oxidase/peroxidase at 37 °C for 35 min, followed by absorbance of the mixture at 500 nm. Starch digestion rate (SDR) was evaluated as a hydrolyzed starch percentage at different times (0, 30, 60, 90, 120, and 180 min). The area under the hydrolysis curve (AUC, 0–180 min) was enumerated. 

#### 4.9.2. α-Glucosidase Inhibition Assay

The percentage of α-glucosidase inhibition activity was measured, as already reported [53]. The assay principle is about measuring the amount of para-nitrophenolate released by para-nitrophenyl glucopyranoside. The hydroethanolic extract of seeds and sprouts was mixed with α-glucosidase (2 U/mL) and incubated at 37 °C for 5 min. Then, 1 mM of para-nitrophenylglucopyranoside was added, dissolved in a phosphate buffer of 50 mM (pH 6.8) to the reaction buffer, and incubated for 20 min at 37 °C. The reaction was shut down by the addition of sodium carbonate (1M). The activity of α-glucosidase was measured at 405 nm. The percentage of inhibition was calculated. 

#### 4.9.3. α-Amylase Inhibition Assay

α-Amylase inhibition was evaluated by mixing starch (2 mg) with 0.5 M Tris-HCl buffer (pH 6.9) and 0.01 M CaCl2, boiling for 5 min, cooling at room temperature, and then incubating for 5 min at 37 °C. Then, the α-amylase (U/mL) was added and incubated again at 37 °C for 10 min. After that, 500 μL 0.1% 3,5-dinitro salicylic acid was added and incubated for 10 min at 100 °C. After cooling, the absorbance was determined at 540 nm for α-amylase inhibition calculation. The percentage of inhibition was calculated. 

### 4.10. Statistical Analyses

The *p* < 0.05 values were used to illustrate the statistical importance among the groups. The statistical analysis was conducted using both SPSS and MS Excel. Replication of each experiment was performed twice. Three to five replicates were used for all assays and each of the replicates corresponded to a group of control plants and Mo-treated plants. The PCA was carried out on R software.

## 5. Conclusions

The results demonstrated the benefits of Mo seed priming of three *Canavalia* species/cultivars to improve growth and N nutrition. Mo enrichment significantly triggered the photosynthetic pigments and enhanced the biomass of sprouts. In CA1, the lipids, fibers, flavonoids, and saponins were significantly increased, and in CA3 a significant rise was observed in phenolics and flavonoids. In CA3, higher differences were observed in N assimilation processes and amino acids as compared to CA1 and CA2, indicating that Mo-mediated impacts were greater in CA3. Further, Mo treatment affected the biosynthesis pathway of phenols, revealed by increased phenolic compounds in the sprouts. The raised levels of phenols and flavonoids were attributed to enhanced antioxidant acidity in CA1 and CA3. Differential patterns revealed that Mo-mediated impacts were not uniform in the *Canavalia* species/cultivars, which might be due to genetic variations and differential uptake. Overall, the findings from this research may facilitate a better understanding of the relationship of Mo application with yield and provide useful information for increasing the nutritional and pharmacological value of *Canavalia* species.

## Figures and Tables

**Table 1:** Effect of molybdenum treatment on the nutritional value of *Canavalia* species/cultivar sprouts. Total lipids (µg/gFW), total proteins (µg/gFW), fibers (µg/gFW), alkaloids (µg/gFW), phenolics (µg/gFW), flavanoids (µg/gFW), saponins (µg/gFW), and glycosides (µg/gFW) were measured. Experiments were executed in triplicate and the data are presented as mean ± standard deviation (SD); Level of significance * *p* < 0.05. Mo, Molybdenum.

	*C. ensiformis var. gladiata* (CA1)		*C. ensiformis var. truncata Ricker* (CA2)		*C. gladiata var. alba Hisauc* (CA3)	
	Control	Mo-Treated	Control	Mo-Treated	Control	Mo-Treated
Total lipids	57.009 ± 5.12	98.085 ± 0.72 *	81.201 ± 7.52	108.250 ± 28.92	92.659 ± 18.44	70.837 ± 3.35
Total proteins	189.607 ± 2.93	219.279 ± 14.73	146.753 ± 40.14	188.859 ± 7.88	120.820 ± 24.16	160.784 ± 14.59
Fibers	9.172 ± 1.65	12.241 ± 0.04 *	14.543 ± 1.04	14.542 ± 3.46	24.364 ± 7.42	18.242 ± 3.75
Alkaloids	2.324 ± 0.77	2.626 ± 0.41	2.669 ± 0.02	2.594 ± 0.47	2.788 ± 1.24	2.437 ± 0.78
Phenolics	1.868 ± 0.11	1.663 ± 0.41	2.323 ± 0.40	2.732 ± 0.22	0.931 ± 0.06	2.630 ± 0.71 *
Flavonoids	0.240 ± 0.00	0.523 ± 0.24 *	0.273 ± 0.07	0.486 ± 0.03	0.132 ± 0.012	0.613 ± 0.13 *
Saponins	1.386 ± 0.23	2.284 ± 0.39 *	2.400 ± 0.32	2.998 ± 0.30	1.420 ± 0.33	2.348 ± 0.30
Glycosides	2.532 ± 0.45	3.712 ± 0.46	3.194 ± 0.37	4.948 ± 0.08	2.708 ± 0.68	3.749 ± 0.09

**Table 2:** Effect of molybdenum treatment on amino acid content (µmol/ gFW) of *Canavalia* species/cultivar sprouts. Experiments were carried out in triplicate and the data are expressed as mean ± standard deviation (S.D); Level of significance, * *p* < 0.05; Mo, Molybdenum.

	*C. ensiformis var. gladiata* (CA1)		*C. ensiformis var. truncata Ricker* (CA2)		*C. gladiata var. alba Hisauc* (CA3)	
	Control	Mo-Treated	Control	Mo-Treated	Control	Mo-Treated
Asparagine	1.22 ± 0.05	1.41 ± 0.06	0.84 ± 0.08 *	1.261 ± 0.03	1.03 ± 0.07	1.7 ± 0.11 *
Glutamine	3.3 ± 0.19	3.98 ± 0.37	2.62 ± 0.21	5.060 ± 0.4 *	3.81 ± 0.22	6.29 ± 0.55 *
Glutamic acid	3.04 ± 0.39	2.42 ± 0.93	1.5 ± 0.23	4.040 ± 0.2 *	2.75 ± 0.22	4.92 ± 0.65
Serine	0.8 ± 0.03	1.13 ± 0.08	0.89 ± 0.05	1.301 ± 0.7	1 ± 0.06	1.15 ± 0.06
Glycine	0.46 ± 0.01	0.77 ± 0.02 *	0.6 ± 0.03	0.6 ± 0.00	0.58 ± 0.05	0.89 ± 0.01 *
Arginin	0.25 ± 0.01	0.34 ± 0.01	0.3 ± 0.01	0.25 ± 0.00	0.32 ± 0.02	0.34 ± 0.03
Alanine	0.49 ± 0.17	0.92 ± 0.05 *	0.49 ± 0.22	0.834 ± 0.01 *	1.5 ± 0.59	1.13 ± 0.7
Proline	0.3 ± 0.17	0.67 ± 0.13 *	0.32 ± 0.23	0.698 ± 0.03 *	1.33 ± 0.19	0.96 ± 0.04
Histidine	0.52 ± 0.02	0.71 ± 0.07	0.41 ± 0.07	0.366 ± 0.08	0.33 ± 0.02	0.58 ± 0.05 *
Valine	0.48 ± 0.03	0.51 ± 0.04	0.29 ± 0.05	0.28 ± 0.0	0.30 ± 0.03	0.46 ± 0.04
Methionine	0.4 ± 0.01	0.5 ± 0.08	0.41 ± 0.01	0.544 ± 0.08	0.42 ± 0.02	0.85 ± 0.05 *
Cystine	0.32 ± 0.08	0.72 ± 0.11 *	0.74 ± 0.07	0.944 ± 0.1 *	0.65 ± 0.08	1.2 ± 0.05 *
Isoleucine	0.41 ± 0.08	1.09 ± 0.11 *	0.72 ± 0.03	0.757 ± 0.2	0.64 ± 0.06	1.35 ± 0.13 *
Leucine	0.66 ± 0.12	1.44 ± 0.14 *	0.75 ± 0.11	0.707 ± 0.11	1.21 ± 0.11	0.9 ± 0.1
Tyrosine	0.33 ± 0.01	0.39 ± 0.02	0.3 ± 0	0.396 ± 0.08	0.28 ± 0.01	0.45 ± 0.0 *
Lysine	0.61 ± 0.03	1 ± 0.2	0.62 ± 0.03	1.075 ± 0.05 *	0.48 ± 0.04	0.91 ± 0.04 *
Threonine	0.55 ± 0.04	0.71 ± 0.02	0.67 ± 0.04	0.76 ± 0.01	0.47 ± 0.03	0.77 ± 0.01 *
Tryptophan	0.2 ± 0.01	0.29 ± 0.02	0.21 ± 0.01	0.38 ± 0.01 *	0.18 ± 0.02	0.4 ± 0.02 *

**Table 3:** Effect of molybdenum treatment on phenolic compounds of *Canavalia* species/cultivar sprouts. Values are expressed as µmol/gFW. Experiments were performed in triplicate and the data are shown as mean ± standard deviation (S.D); Asterisks (*) show the level of significance, * *p* < 0.05; Mo, Molybdenum.

	*C. ensiformis var. gladiata* (CA1)		*C. ensiformis var. truncata Ricker* (CA2)		*C. gladiata var. alba Hisauc* (CA3)	
	Control	Mo-Treated	Control	Mo-Treated	Control	Mo-Treated
Gallic acid	0.88 ± 0.03	1.44 ± 0.1 *	0.72 ± 0.11	0.58 ± 0.1	1.2 ± 0.09	0.63 ± 0.22 *
Caffeic acid	1.71 ± 0.3	2.36 ± 0.34	1.782 ± 0.38	3.15 ± 0.26 *	3.218 ± 0.18	3.27 ± 0.37
p-Coumaric acid	2.3 ± 0.31	3.44 ± 0.41 *	2.22 ± 0.44	3.32 ± 0.27 *	3.935 ± 0.21	3.42 ± 0.22
Chicoric acid	0.79 ± 0.03	0.55 ± 0.18	0.83 ± 0.04	1.34 ± 0.13 *	0.93 ± 0.07	1.22 ± 0.13 *
Rosmarinic acid	0.26 ± 0.01	0.37 ± 0.03 *	0.27 ± 0.05	0.41 ± 0.02 *	0.483 ± 0.06	0.48 ± 0.03
Protocatechuic acid	1.52 ± 0.21	1.48 ± 0.19	1.53 ± 0.1	1.60 ± 0.12	2.24 ± 0.28	1.94 ± 0.1
Quercetin	0.114 ± 0.02	0.14 ± 0.03	0.09 ± 0.01	0.105 ± 0.02	0.131 ± 0.02	0.141 ± 0.02
Naringenin	0.15 ± 0.01	0.98 ± 0.02	0.78 ± 0.01	1.56 ± 0.01 *	1.58 ± 0.01	1.23 ± 0.01
Kaempferol	0.72 ± 0.02	0.615 ± 0.1	1.10 ± 0.16	1.104 ± 0.17	1.094 ± 0.11	1.91 ± 0.11 *
Luteolin	0.48 ± 0.23	0.34 ± 0.22	0.426 ± 0.11	0.42 ± 0.18	0.48 ± 0.16	0.40 ± 0.2
Apigenin	0.35 ± 0.12	0.3 ± 0.03	0.004 ± 0.03	0.015 ± 0.01 *	0.017 ± 0.03	0.015 ± 0.01
Naringenin	0.59 ± 0.02	1.272 ± 0 *	0.861 ± 0.2	0.97 ± 0	1.66 ± 0	1.87 ± 0.1
Rutin	0.945 ± 0	1.10 ± 0.014	0.108 ± 0.22	0.104 ± 0.08	1.44 ± 0.2	1.47 ± 0.28
Chlorogenic acid	0.08 ± 0.06	1.41 ± 0.28 *	0.84 ± 0.45	1.04 ± 0.24	1.04 ± 0.36	1.71 ± 0.29 *

## Data Availability

Data presented in this study are available on reasonable request.
